# Psychogenic Dysphagia in an Elderly: A Case of Eating Disorder Due to Trauma and Grief

**DOI:** 10.7759/cureus.47137

**Published:** 2023-10-16

**Authors:** Hana A Alzuabi, Atheer A Altamimi, Awad Al Harbi, Sultan M Al Shahrani, Abdullah Al Faris

**Affiliations:** 1 Neurology, Princess Nourah Bint Abdulrahman University, Riyadh, SAU; 2 Neurosciences, King Abdullah bin Abdulaziz University Hospital, Princess Nourah Bint Abdulrahman University, Riyadh, SAU; 3 Psychiatry, King Abdullah bin Abdulaziz University Hospital, Princess Nourah Bint Abdulrahman University, Riyadh, SAU; 4 Rehabilitation Services, King Abdullah bin Abdulaziz University Hospital, Princess Nourah Bint Abdulrahman University, Riyadh, SAU

**Keywords:** swallowing problem, geriatric, dysphagia, eating disorder, psychogenic dysphagia

## Abstract

Psychogenic dysphagia is a swallowing condition caused by psychological factors rather than structural or physiological disorders such as neurological impairments or physical abnormalities. This condition has a significant impact on a patient's quality of life. Patients should undergo a thorough swallowing evaluation with the involvement of a multidisciplinary team as early intervention leads to satisfactory outcomes. This report presents a case of a 62-year-old female who had severe malnutrition due to psychogenic dysphagia. We evaluated organic and other functional causes of dysphagia, but no abnormalities were detected.

## Introduction

Dysphagia is one of the most prevalent disorders with a prevalence of 10% to 22%, and this number rises with advanced age to reach up to 40% in people aged over 60 [1]. Although the mechanisms that contribute to the formation and maintenance of functional gastrointestinal disorders such as dysphagia are diverse and multidimensional [2,3], multiple studies have revealed favorable links between gastrointestinal disorders and psychological disorders [4,5]. Psychogenic dysphagia is a swallowing condition caused by psychological factors rather than structural or physiological disorders such as neurological impairments or physical abnormalities [6]. It might manifest suddenly or gradually, accompanied by discomfort or fear of swallowing specific food, liquid, or pills and may result in malnutrition and weight loss [7,8]. This condition can be stressful for the patients, resulting in a reduction in the patient's quality of life [8]. According to several studies, approximately 45-70% of people with dysphagia have a comorbidity of anxiety disorders, which are accompanied by psychosocial conditions such as divorce, unemployment, post-traumatic stress disorder, and others [9]. Psychogenic dysphagia is a rare and poorly understood swallowing disorder without a structural or organic disease. In a patient with suspected psychogenic dysphagia, a thorough swallowing evaluation is required with a multidisciplinary team [7].

Only a few case reports/series and review articles are currently available on this uncommon disorder [10]. Although physicians frequently encounter patients with psychogenic dysphagia during routine clinical practice, there is almost no data addressing this condition in Saudi Arabia. In this report, we describe a case of a female patient who is presenting with severe swallowing problems, malnutrition, and significant weight loss due to psychogenic dysphagia.

## Case presentation

A 62-year-old female non-smoker with a known case of gastroesophageal reflux disease and on regular medications (on omeprazole 40 mg) due to Helicobacter pylori was brought by her family to the ED at King Abdullah bin Abdulaziz University Hospital (KAAUH), Saudi Arabia, in December 2022. Her compline was worsening shortness of breath for 23 days before presenting to the ED. She has no previous history of shortness of breath, associated with cough, generalized fatigue, fever (38 ° documented at home), and palpitation. She was admitted to the hospital and managed as a case of aspirational pneumonia (treated with Tazocin 4.5 mg).

Upon further investigation by the patient’s daughters, it was found that the patient has a history of dysphagia. Her dysphagia started almost seven years ago and developed as a progressive swallowing problem, particularly with solid food. No investigations were done at that time, and it was managed by the patient by eliminating anything that could cause difficulty swallowing. She denied ingestion of a caustic agent or foreign body prior to the onset of her symptoms. She reported no other gastrointestinal complaints.

Her condition worsened over the last year and progressed from difficulty in swallowing solid to semi-solid and then to liquid where she reported choking with her own saliva sometimes. She complained of loss of appetite as well; therefore, her health continued to decline, resulting in a drastic weight loss of up to 20 kg over the span of six months (the patient's initial weight was unknown, and during the examination, the patient's weight was 33.2 kg, BMI 13 Kg/m2).

In addition, it was found that she has a lifetime history of generalized anxiety disorder with multiple complicated griefs that started with the sudden loss of her favorite loveable youngest son as described by the daughters in 2016. Afterward, there were multiple sudden deaths of close family members over the last few years.

Meanwhile, the timing of dysphagia worsening and weight loss was comparable to the timing of frequently complicated grief. Multiple invasive investigations were done in different institutes. However, no organic explanations, such as a severe case of dysphagia and progressive weight loss, were identified.

Subjectively, the patient acknowledged her fearfulness, and chest tightness with sleep disturbance over the last year and it was getting worse in this current admission. Her family history was remarkable for lung cancer on her brother's side, otherwise, it was not contributory.

## Discussion

Evaluation

During her hospital course, the patient continued on enteral feeding via nasogastric tube, and she was evaluated by a multidisciplinary team. The findings of each assessment will be discussed in further sections.

**Figure 1:**
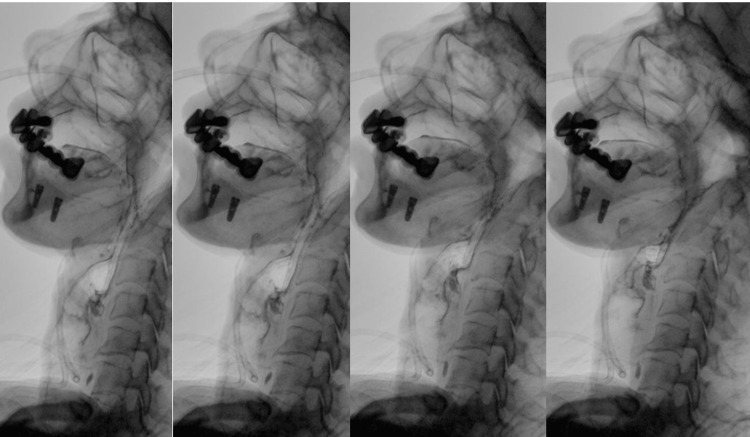
Modified barium swallow study Shows silent aspiration

A bedside swallowing assessment showed severe pharyngeal dysphagia. In the oral stage, there was reduced oral strength, decreased lip sealing, reduced tongue manipulations, no anterior spillage, and no residues in the anterior and lateral sulcus. The pharyngeal stage showed a decreased laryngeal elevation and hyolaryngeal excursion, repeated swallows seven times for 1 ml, slow swallow, and clear signs of aspiration with thin liquid (1 ml) characterized by an audible swallow and wet voice. A modified barium swallow study was requested, and the results confirmed that the patient has moderate to severe pharyngeal dysphasia characterized by aspiration with all consistencies (Figure 1).

**Figure 2:**
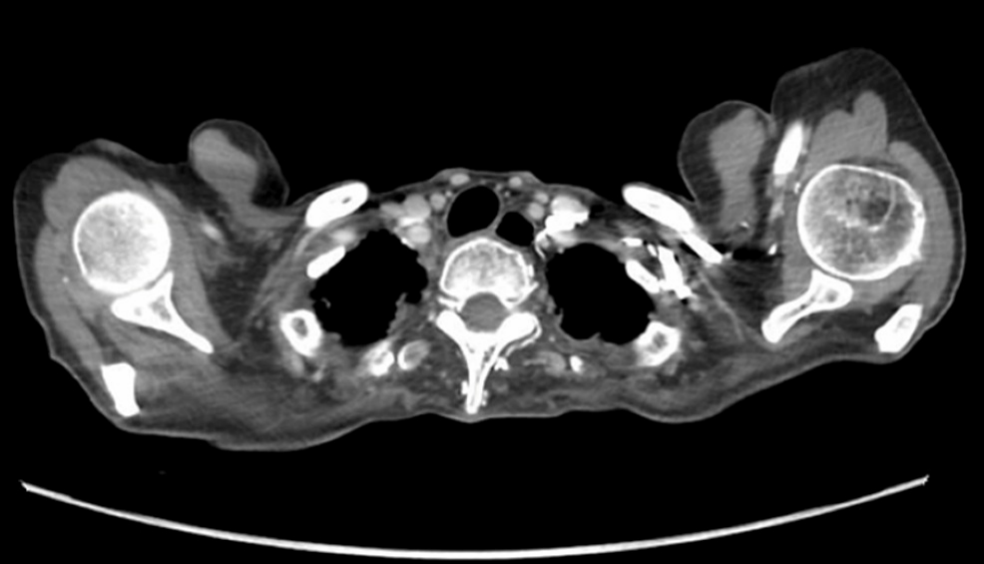
Head and neck CT scan with IV contrast Mucus plugging seen in the right lower lobe bronchus with small subpleural ground-glass opacities in the lower lobes bilaterally could be aspiration/infection.

The otorhinolaryngology team requested a CT which showed a dilated upper esophagus with no mediastinal mass compressing the esophagus identified. A bilateral small hypodense thyroid nodule was noted (Figure 2).

The patient was found to be cachexic; therefore, a PET scan was requested to rule out occult malignancies, and it was unremarkable. A bronchoscopy was done and showed mucus plugging which was cleared from the right middle lobe and right left lobe erythema all over which indicates chronic bronchitis, bronchomalacia, and no mass or foreign bodies.

Neurological causes were ruled out by nerve conduction studies/electromyography which showed no evidence of motor neuron disease or neuromuscular junction disorder. MRI brain and cervical spine showed no evidence of pathology that could be the cause of her swallowing difficulty. Both acetylcholine receptor antibody and anti-MUSK antibody were negative.

Esophagogastroduodenoscopy was performed as well and showed a small upper esophageal submucosal lesion 10 cm below the upper esophagus which should not cause dysphagia.

Discussion

According to the Diagnostic and Statistical Manual of Mental Disorders, Fifth Edition (DSM-5), psychogenic dysphagia diagnostic criteria and guidelines are listed under the category of avoidant/restrictive food intake disorder (ARFID) [9]. This patient displayed symptoms of psychogenic dysphagia disorder. Her symptoms began to occur shortly after her son passed away. She began to experience dysphagia, which gradually went from solid to liquid. Her symptoms were associated with abnormal grief in the background of anxiety disorder as well. Her symptoms were associated with loss of appetite and weight loss. These symptoms can indicate a broad spectrum of other conditions including structural abnormalities, neuromuscular diseases, gastrointestinal conditions, metabolic and inflammatory problems, cardiopulmonary problems, and iatrogenic causes [11,12]. Moreover, criterion D of the ARFID specifies that the disorder cannot be explained by a concurrent medical condition [13]. Therefore, psychogenic dysphagia can only be diagnosed after excluding all other possible medical conditions. Hence, our patient underwent an extensive workup which revealed no structural or functional causes. After the modified barium swallow study confirmed the diagnosis of severe pharyngeal dysphagia, we further evaluated the patient to rule out structural causes. The results of the CT showed dilation in the upper esophagus with no mediastinal compressing mass. EGD revealed a submucosal lesion which is too small to cause any effect. A PET scan was performed, but no related abnormalities were found.

Furthermore, this patient experienced a traumatic life event with multiple deaths of her family members which matched the course of her symptoms. These multiple complicated griefs might be the trigger of her dysphagia as there have been several reports of induced eating disorders after abnormal grief reactions [14]. Therefore, in the absence of any structural or functional causes, the diagnosis of psychogenic dysphagia was confirmed, and the patient was referred to a psychiatrist for assessment. The psychiatrist assessed the patient. He found depressive symptoms and grief reaction symptoms because she had several deaths in her family. She reported that she lost her brother recently about three months prior to her current admission. She lost her mother and her elder brother about five years ago. She also lost her son seven years ago because of a road traffic accident. Other possible causes of psychogenic dysphagia were ruled out including anxiety disorders, emotional stress [15], and current or previous use of antipsychotic medications [16-19]. She was prescribed mirtazapine, which helped with depression but did not improve psychogenic dysphagia. She has follow-ups in the psychiatry clinic.

In psychogenic dysphagia, early identification and treatment are crucial because it is highly distressing and likely to cause weight loss and malnutrition if left untreated.

Many known therapeutic modalities include antidepressants, psychoeducation, hypnosis, cognitive behavioral therapy, eye movement desensitization and reprocessing, and exposure therapy. Kim et al. suggested considering electroconvulsive therapy to be a therapeutic option if other methods failed as he reported a case with complete resolution of psychogenic dysphagia [20].

## Conclusions

Psychogenic dysphagia has a significant impact on a patient's quality of life. Patients should undergo a thorough swallowing evaluation with the involvement of a multidisciplinary team as early intervention leads to satisfactory outcomes. We presented a 62-year-old female patient who had severe malnutrition due to trauma and grief. Based on her past history, it was found that the patient experienced multiple complicated griefs, and her physical and supporting examinations were found to be in normal condition. Numerous invasive tests were performed at several institutes, but no organic causes for her severe dysphagia and continuing weight loss were found. Accordingly, the patient was diagnosed with psychogenic dysphagia, which falls under the DSM-5 category of ARFID.
